# Integrated Transcriptome and Metabolome Analyses Provide Insights into the Coloring Mechanism of Dark-red and Yellow Fruits in Chinese Cherry [*Cerasus pseudocerasus* (Lindl.) G. Don]

**DOI:** 10.3390/ijms24043471

**Published:** 2023-02-09

**Authors:** Yan Wang, Zhiyi Wang, Jing Zhang, Zhenshan Liu, Hao Wang, Hongxia Tu, Jingting Zhou, Xirui Luo, Qing Chen, Wen He, Shaofeng Yang, Mengyao Li, Yuanxiu Lin, Yunting Zhang, Yong Zhang, Ya Luo, Haoru Tang, Xiaorong Wang

**Affiliations:** 1 College of Horticulture, Sichuan Agricultural University, Chengdu 611130, China; 2 Institute of Pomology and Olericulture, Sichuan Agricultural University, Chengdu 611130, China

**Keywords:** *Cerasus pseudocerasus* (Lindl.) G. Don, transcriptome, metabolome, anthocyanin, candidate gene, transcription factor

## Abstract

Chinese cherry [*Cerasus pseudocerasus* (Lindl.) G. Don] is an important fruit tree from China that has excellent ornamental, economic, and nutritional values with various colors. The dark-red or red coloration of fruit, an attractive trait for consumers, is determined by anthocyanin pigmentation. In this study, the coloring patterns during fruit development in dark-red and yellow Chinese cherry fruits were firstly illustrated by integrated transcriptome and widely-targeted metabolome analyses. Anthocyanin accumulation in dark-red fruits was significantly higher compared with yellow fruits from the color conversion period, being positively correlated to the color ratio. Based on transcriptome analysis, eight structural genes (*CpCHS*, *CpCHI*, *CpF3H*, *CpF3’H*, *CpDFR*, *CpANS*, *CpUFGT*, and *CpGST*) were significantly upregulated in dark-red fruits from the color conversion period, especially *CpANS*, *CpUFGT*, and *CpGST*. On contrary, the expression level of *CpLAR* were considerably higher in yellow fruits than in dark-red fruits, especially at the early stage. Eight regulatory genes (*CpMYB4*, *CpMYB10*, *CpMYB20*, *CpMYB306*, *bHLH1*, *CpNAC10*, *CpERF106*, and *CpbZIP4*) were also identified as determinants of fruit color in Chinese cherry. Liquid chromatography-tandem mass spectrometry identified 33 and 3 differential expressed metabolites related to anthocyanins and procyanidins between mature dark-red and yellow fruits. Cyanidin-3-*O*-rutinoside was the predominant anthocyanin compound in both fruits, while it was 6.23-fold higher in dark-red than in yellow fruits. More accumulated flavanol and procyanidin contents resulted in less anthocyanin content in flavonoid pathway in yellow fruits due to the higher expression level of *CpLAR*. These findings can help understand the coloring mechanism of dark-red and yellow fruits in Chinese cherry, and provide genetic basis for breeding new cultivars.

## 1. Introduction

Anthocyanins and proanthocyanidins (PAs), known as flavonoids, belong to the group of the ubiquitous secondary metabolites. Anthocyanins are a group of important natural water-soluble pigments that commonly produce red/purple/blue colors to flowers and fruits of plants [1,2]. They have shown health-promoting properties, including antioxidant activity, cholesterol decomposition, visual acuity, and prevention of cardiovascular disease in humans [3]. In nature, anthocyanins existed as glycosides of polyhydroxy and polymethoxy derivatives mainly including cyanidin, pelargonidin, peonidin, delphinidin, malvidin, and petunidin [1,4]. The composition and proportion of anthocyanins determine the coloration of plant tissues. PAs are essential taste factors affecting astringency and bitterness of fruits, which are also considered as important determinants of fruit quality [5].

Anthocyanins and PAs are synthesized by multiple enzyme-encoding structural genes via the flavonoid pathway. The phenylalanine forms anthocyanins, being catalyzed by phenylalanine ammonia-lyase (*PAL*), cinnamate 4-hydroxylase (*C4H*), 4-coumarate-CoA ligase (*4CL*), early biosynthesis genes (EBGs) (chalcone synthase (*CHS*), chalcone isomerase (*CHI*), flavanone 3-hydroxylase (*F3H*), flavonoid 3’-hydroxylase (*F3’H*), flavonoid 3’,5’-hydroxylase (*F3’5’H*)), and late biosynthesis genes (LBGs) (dihydroflavonol-4-reductase (*DFR*), anthocyanidin synthase/leucoanthocyanidin dioxygenase (*ANS*/*LDOX*), UDP-glucose: flavonoid-3-*O*-glucosyl-transferase (*UFGT*), and glutathione S-transferase (*GST*)) [1,6,7,8,9]. Leucoanthocyanidins and anthocyanins are further catalyzed by leucoanthocyanidin reductase (*LAR*) and anthocyanidin reductase (*ANR*) to form catechin and epicatechin, finally forming PAs [5]. In addition, all these structural genes are regulated by a MYB-bHLH-WD40 (MBW) complex at the transcriptional level [6,10,11,12].

Chinese cherry [*Cerasus pseudocerasus* (Lindl.) G.Don], belonging to the genus *Cerasus* of the family Rosaceae, is an economically important tetraploid fruiting cherry species [13,14]. It is native to China and has been widely cultivated across China as an important deciduous fruit with high economic and ornamental values [15]. Recently, cherry cultivation has been developing rapidly in China and has increasingly contributed to poverty alleviation and rural revitalization. Because of its diverse adaptability to various environments, Chinese cherry has not only been widely used as rootstock for cherry varieties, but it is also an excellent gene donor for intraspecific hybridization breeding program [16,17,18]. The fruits possess many valuable traits, including their various colors, unique taste, and abundant vitamins, fiber, minerals, and antioxidant compounds for healthy diets [15,19]. The majority of Chinese cherry germplasms have red, dark-red or orange-red fruit color, with a small number of accessions with black purple or yellow fruit color [20].

In cherry fruits, the difference between red and yellow color of the fruit peel and flesh is mainly dependent on the accumulation of anthocyanins. However, the component and content of specific anthocyanins vary with different cherry varieties [21]. Seven anthocyanins have been detected in four cherry species [22]. Cyanidin 3-rutinoside and cyanidin 3-glucosyl-rutinoside were the major components in both *C. pseudocerasus* and *C. vulgaris* [22], and cyanidin 3-rutinoside was predominant in *C. avium* [22,23,24,25]. Pelargonidin 3-*O*-glucoside, cyanidin 3-*O*-rutinoside, and pelargonidin 3-*O*-rutinoside were the chief compounds in the red *C. tomentosa* compared with the white fruit [26]. In *C. avium* and *C. tomentosa*, a large number of structural and regulatory genes involved in anthocyanin biosynthesis, transport and degradation pathway, have been identified by previous studies [26,27,28,29]. It is worth noting that the major anthocyanin component and the related genes/transcription factors (TFs) regulating anthocyanin biosynthesis varied from different cherry species. Therefore, it is necessary to explore the key candidate genes and potential molecular mechanism regulating anthocyanin levels of Chinese cherry fruit with different colors.

With the recent advancements in transcriptome and metabolome, the integrative analysis has provide an effective approach to illustrate the metabolic pathways and regulatory genes in fruit crops, such as sweet cherry [30], tomentosa cherry [26], strawberry [11], and longan [31]. In this study, integrative analysis of transcriptome and metabolome data were conducted to (i) analyze the color differences between dark-red and yellow Chinese cherry fruits, (ii) identify the differential expressed genes associated with anthocyanin biosynthesis during fruit development, and (iii) identify the key differential expressed metabolites related to anthocyanins and proanthocyanidins between mature dark-red and yellow fruits. These results provide insights for the identification of metabolites and candidate genes involved in anthocyanin formation and color change between dark-red and yellow Chinese cherry fruits, which lays a molecular foundation for color improvement and breeding program in the future.

## 2. Results

### 2.1. Color Phenotypic Characterization of Chinese Cherry during Fruit Development

**Figure 1:**
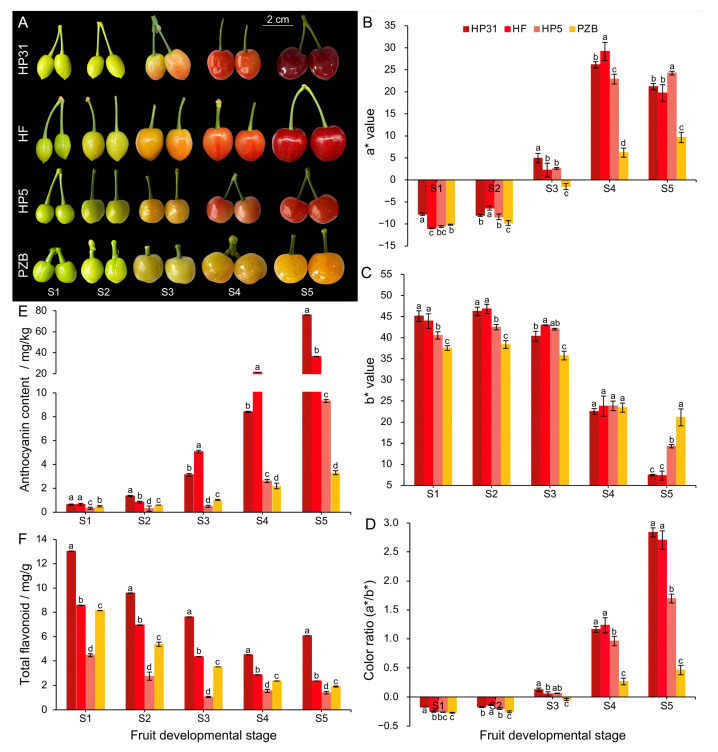
Color phenotypic characterization of Chinese cherry during fruit development. (**A**) Fruit phenotypes during development in dark-red and yellow fruits. (**B**) The a* values. (**C**) The b* values. (**D**) Color ratio (a*/b*). The a* coordinate represents the red (positive)-to-green (negative) scale, the b* coordinate represents the yellow (positive)-to-blue (negative) scale. Color ratio (a*/b*) represents the comprehensive color index [32]. (**E**) Total anthocyanin content. (**F**) Total flavonoids content. Error bars indicate ± standard deviation (SD) from three independent biological replicates. The lower case letters indicate significant difference at 0.05 level.

Phenotypic observation at various developmental stages revealed significant differences in fruit peel color among the four Chinese cherry accessions. ‘HP31’, ‘HF’, and ‘HP5’ turned pink blush at S3 stage and the whole fruit (peel and flesh) color became dark-red, red, or light-red at S5 stage; whereas ‘PZB’ remained yellow throughout the corresponding developmental stage (Figure 1A). The a*, b*, and a*/b* ratio were also considered as indicators of fruit color (Figure 1B–D). The a*/b* ratio was significantly higher in dark-red fruits than in yellow fruits from the S3 stage, reaching the most significant differences at S5 stage compared with yellow fruits (Figure 1D). Consistent with the color change in the fruit, anthocyanin biosynthesis started at S2 stage, significant differences in anthocyanin content among the four accessions appeared at S3 stage, and anthocyanins accumulated in large quantities by S5 stage (Figure 1E). The total anthocyanin content was 23-fold higher in ‘HP31’ (75.96 mg/kg FW) than in ‘PZB’ (3.31 mg/kg FW) fruits at mature stage (S5), indicating that the anthocyanin accumulation was enhanced in the dark-red Chinese cherry fruits. Significant higher anthocyanin content was also detected in red fruits ‘HF’ and ‘HP5’ than that in ‘PZB’ (Figure 1E). The green fruits (S1) showed the highest total flavonoids content for the all four accessions, and decreased to at red stage (S4) for ‘HP31’ and increased to 6.08 mg/g at dark-red stage (S5) (Figure 1F). At mature stage, the highest and the lowest contents of flavonoids were detected in ‘HP31’ and ‘HP5’, respectively (Figure 1F).

### 2.2. Differentially Expressed Genes Analysis between Dark-red and Yellow Fruits

To identify the genes related to the fruit color formation between dark-red and yellow fruits, the fruits at S1-S5 stage for four accessions were subjected to RNA-seq. The raw transcriptome sequences from 60 samples have been submitted to the CNGB database under project number CNP0003682. After filtering the raw data, 2,800,020,582 clean reads were obtained, ranging from 41,582,094 to 48,104,774 per sample. The GC content was more than 46.54%, and the Q20 values ranged from 93.86% to 97.36%. The comparison rate exceeded 85.65% with the genome of Chinese cherry (unpublished data) as the reference genome (Supplementary Table S1).

We compared the transcriptome profiles of dark-red and yellow fruits to identify differentially expressed genes (DEGs) during fruit development. There were more DEGs between dark-red and yellow fruits than that during fruit development of the same accession (Supplementary Figure S1A). For each developmental stage between dark-red and yellow fruits, the down-regulated DEGs were more abundant than the up-regulated DEGs at S1, S2, and S4 stages, but the up-regulated DEGs were more abundant than the down-regulated DEGs at S3 and S5 stages (Supplementary Figure S1A). At total of 477, 591, 1146, 1682, and 1022 common DEGs were identified between dark-red and yellow fruits at S1-S5 stages, respectively (Supplementary Figure S1B). Within each accession, the gene expressions differences were the most significant from stage S2 to S4. A total of 102, 243, 578, and 115 common DEGs were identified in the S1 vs. S2, S2 vs. S3, S3 vs. S4, S4 vs. S5 comparison groups within dark-red fruits (Supplementary Figure S1C). A total of 1047, 2833, 2027, and 1936 DEGs were identified in the S1 vs. S2, S2 vs. S3, S3 vs. S4, S4 vs. S5 comparison groups in yellow fruits (Supplementary Figure S1A).

Kyoto Encyclopedia of Genes and Genomes (KEGG) analysis provided additional information about the enriched biological pathways, including “metabolism”, “biosynthesis of other secondary metabolites”, “starch and sucrose metabolism”, “amino acid metabolism”, and “flavonoid biosynthesis”, and so on (Supplementary Figure S2). Based on the statistical significance criterion for multiple testing correlation (correlated *p*-value), “metabolism”, “biosynthesis of other secondary metabolites”, “flavonoid biosynthesis”, and “transporters” were significantly enriched at S1-S5 stages between dark-red and yellow fruits (Supplementary Figure S3). Among them, structural genes such as *CHS*, *CHI*, *DFR*, *LAR*, and *ANR* were screened between dark-red and yellow fruits. During developmental stage S2-S4 within dark-red fruits, *C4H*, *CHS*, *CHI*, *F3’H*, *DFR*, and *UFGT* were enriched in the flavonoid biosynthesis pathway (Supplementary Figure S4). In yellow fruits, *C4H*, *FLS*, *DFR*, *LAR*, *ANR*, and *UFGT* were enriched in S2 vs. S3 comparison, and *CHI*, *LAR,* and *UFGT* were identified in S3 vs. S4 comparison (Supplementary Figure S5). These results suggested obvious difference in anthocyanin biosynthesis between dark-red and yellow fruits, especially during color conversion period.

### 2.3. Weight Gene Co-Expression Network Association Analysis

**Figure 2:**
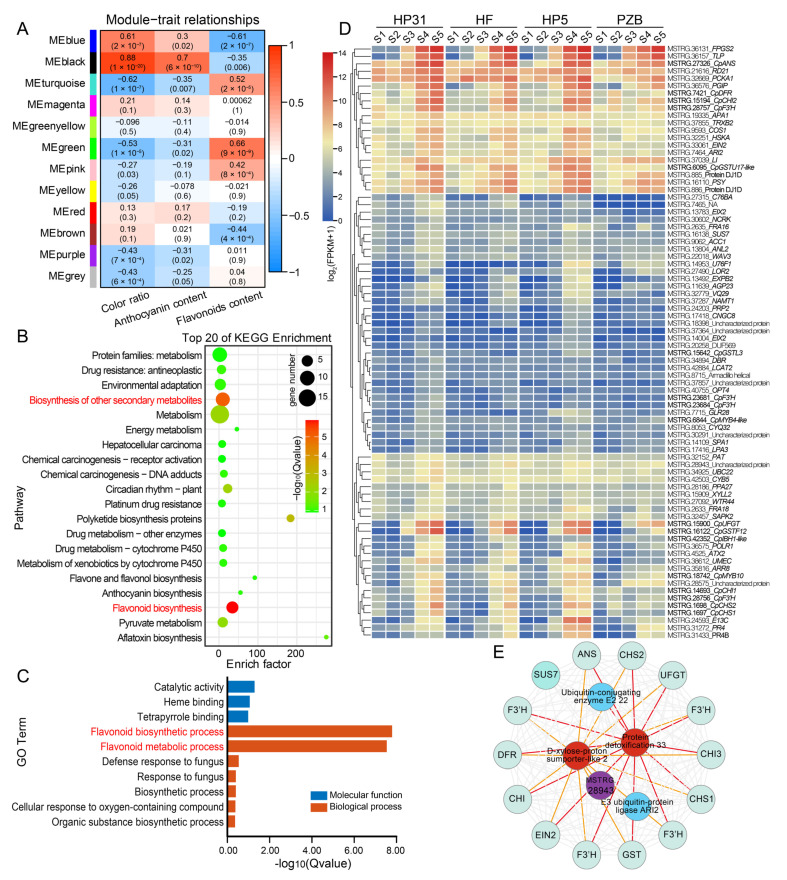
WGCNA recognized gene networks and key candidate genes associated with anthocyanin biosynthesis during fruit development of Chinese cherry. (**A**) Module–trait relationships based on Pearson correlations. The color key from blue to red represents *r^2^* values from −1 to 1. (**B**) KEGG enrichment analysis of with the top 20 KEGG pathways in the genes in the MEblack module. (**C**) GO enrichment analysis of genes in the MEblack module. (**D**) Heatmap of the expression level of genes in the MEblack module. The color scale of the heatmap represents expression levels as log_2_(FPKM + 1). (**E**) Genes whose expression was highly correlated in the MEblack module.

The weighted co-expression network analysis (WGCNA) was conducted based on the normalized expression data for 46,058 genes from all 60 samples. After filtering, 11,515 genes were retained and classified into 12 distinct gene modules (Supplementary Figure S6). Module–trait relationship analysis revealed that six, two and six modules were significantly (*p* < 0.01) correlated with color ratio, anthocyanin content and flavonoid content, respectively (Figure 2A). Among these modules, the MEblack module of 80 genes were highly positively correlated with both color ratio (*r*^2^ = 0.88, *p* = 1 × 10^−20^) and anthocyanin content (*r*^2^ = 0.70, *p* = 6 × 10^−10^) (Figure 2A). The KEGG pathway enrichment analysis revealed that the MEblack module genes were significantly enriched in “flavonoid biosynthesis” (ko00941) and “biosynthesis of other secondary metabolites” (ko09110) (Figure 2B). Gene ontology (GO) enrichment analysis showed that they were significantly enriched in molecular function and biological process category, mainly involved in “flavonoid metabolic process” (GO: 0009812) and “flavonoid biosynthetic process” (GO: 0009813) (Figure 2C).

The hierarchical clustering heatmap was constructed based on FPKM (fragments per kilobase per million fragments) values in each sample, which illustrated the expression patterns of 80 genes in MEblack module among four accessions at five developmental stages (Figure 2D). This module harbored most of structural genes involved in anthocyanin biosynthesis and transport pathway, such as *CHS*, *CHI*, *F3’H*, *DFR*, *ANS*, *UFGT*, and *GST*. Moreover, we annotated two reported MYB homologs involved in anthocyanin biosynthesis in other species. MSTRG.6844 and MSTRG.18742 encode TFs homologous to *MYB4*-like and *MYB10* from *Prunus avium*, and they are related to anthocyanin biosynthesis [29,33]. As an important part of the MBW transcription factor complex regulating the anthocyanin biosynthesis, a *bHLH1* gene (MSTRG.42352), homologous to bHLH from *Arabidopsis thaliana* [34] was also identified (Figure 2D). Hub genes were screened in the MEblack module (Figure 2E), including a protein detoxification 33 (MSTRG.7465), D-xylose-proton symporter-like 2 (MSTRG.15909), MSTRG.28943, ubiquitin-conjugating enzyme E2 22 (MSTRG.34925), probable E3 ubiquitin-protein ligase ARI2 (MSTRG.7464) (Supplementary Table S2).

### 2.4. Expression of Genes and TFs Related to Anthocyanins Biosynthesis

**Figure 3:**
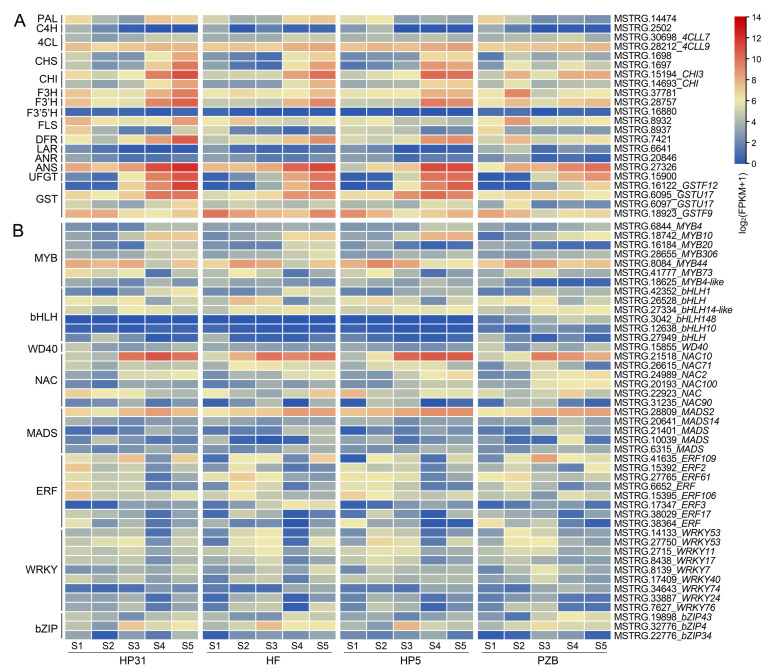
Expression heatmap of structural genes (**A**) and transcription factors (**B**) associated with anthocyanin biosynthesis. The heatmap represents the expression of corresponding genes in Chinese cherry fruits, and from blue to red in the heatmap indicates the expression levels of genes ranging from low to high. The color scale of the heatmap represents expression levels as log_2_(FPKM + 1).

To further investigate the regulatory mechanism underlying anthocyanin accumulation in Chinese cherry fruit, we focused on the 80 MEblack module genes and other structural genes involved in the flavonoid biosynthesis pathway (Figure 3A, Supplementary Table S3). As for the expression levels, no significant differences were detected in the three genes (*PAL*, *C4H,* and *4CL*) involved in phenylpropanoid pathway between dark-red and yellow fruits. The EBGs (*CHS*, *CHI*, *F3H*, and *F3’H*) and LBGs (*DFR*, *ANS*, and *UFGT*) were significantly up-regulated in the dark-red fruits, especially from S3 to S5 stages (Figure 3A), consistent with the high anthocyanin content in dark-red fruits (Figure 1). GST genes was proposed to be involved in the anthocyanin transport, which showed 3.33~4.75-fold higher log_2_fold change values at mature stage in dark-red fruits, being the most significant DEG in the anthocyanin accumulation process (Supplementary Table S3). Interestingly, the expression of *LAR* was up-regulated in yellow fruits especially at the early stage (S1–S2), although their levels were relatively low compared with other genes (Supplementary Table S3). This might imply that more procyanidin content was accumulated in yellow fruits.

It is widely known that anthocyanin biosynthesis was primarily regulated by the MBW protein complex and other TFs. Two TFs, *MYB10* and *bHLH1*, selected from both DEGs and WGCNA, were significantly up-regulated in the dark-red fruits, especially at the later stages (Figure 3B). In addition to them, we also obtained 6 MYB, 5 bHLH, and 1 WD40 in Chinese cherry fruits (Figure 3B, Supplementary Table S3). According to transcriptional levels of candidate genes, 5 MYB and 3 bHLH were upregulated, while *MYB4*, *bHLH148, bHLH10*, and *WD40* were down-regulated in dark-red fruits. Consistent with previous studies [35,36,37,38,39,40,41], other TFs including 6 NAC, 5 MADS, 8 ERF, 9 WRKY, and 3 bZIP were also selected from the DEGs (Figure 3B). These TFs may also exert an effect on or participate in the regulation of structural and regulatory genes in anthocyanin biosynthesis.

### 2.5. RT-qPCR Validation

**Figure 4:**
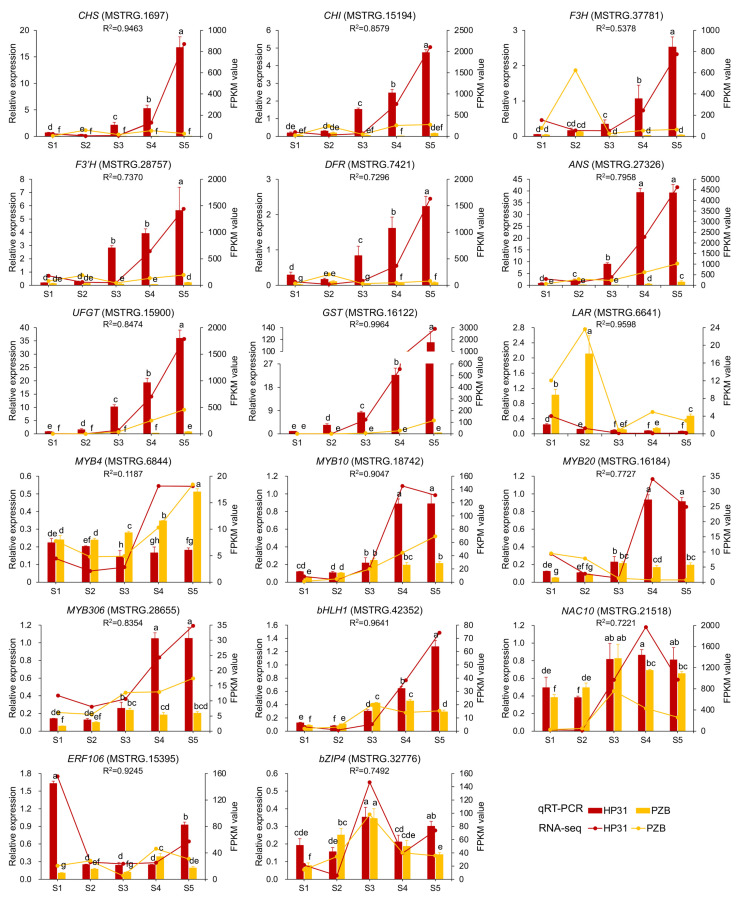
Expression patterns of candidate genes related to anthocyanin accumulation in Chinese cherry during fruit development. Error bars indicated ± SD from three independent replicates. The lower case letter indicated significant difference at 0.05 level for the relative expression level. The expression levels of these genes in ‘HF’ and ‘HP5’ were not shown in this figure.

To verify the reliability of RNA-seq data, real-time PCR was performed to determine the expression levels of seventeen genes (Figure 4). During fruit development, their expression levels gradually upregulated from S1 to S2 stage and dramatically increased from S3 stage, then reached the highest at S5 stage in dark-red fruits. On the contrary, the expression levels slightly up-regulated from S1 to S2 stage, while significantly down-regulated at S3 stage and recovered slightly at S4 and S5 stages in yellow fruits. *CpGST* and *CpANS* showed the highest expression level at the later stages for dark-red fruits (Figure 4). At mature stage, the most significantly different genes were *CpGST*, *CpF3H,* and *CpUFGT*, with about 8.14-, 6.08-, and 5.50-fold log_2_fold change values in the ‘PZB’ vs. ‘HP31’ comparison, respectively. However, *CpLAR* revealed distinct expression profile, which was continuously decreasing during the fruit development in dark-red fruits, while it kept significant higher level in yellow fruits especially at the early stage (Figure 4). The TFs, *CpMYB10*, *CpMYB20*, *CpMYB306*, *CpbHLH1*, *CpNAC10*, and *CpERF106* revealed significantly higher expression levels in dark-red than in yellow fruits, especially at S4 and S5 stages (Figure 4). By contrast, the expression level of *CpMYB4* was significant lower in dark-red than in yellow fruits, suggesting its negative role in anthocyanin biosynthesis (Figure 4). In addition, the expression levels of TFs in different tissues illustrated that these TFs revealed much higher expression levels in fruits and red flower bud than that in root, stem and leaf (Supplementary Figure S7). Finally, the correlation analyses exhibited significant correlation coefficients ranging from 0.7221 to 0.9964 (except for *CpF3H*, R^2^ = 0.5378; *CpMYB4*, R^2^ = 0.1187) between RNA-seq and RT-qPCR (Figure 4), supporting the accuracy of the transcriptome data.

### 2.6. Comparison of Metabolites between Dark-red and Yellow Fruits

**Figure 5:**
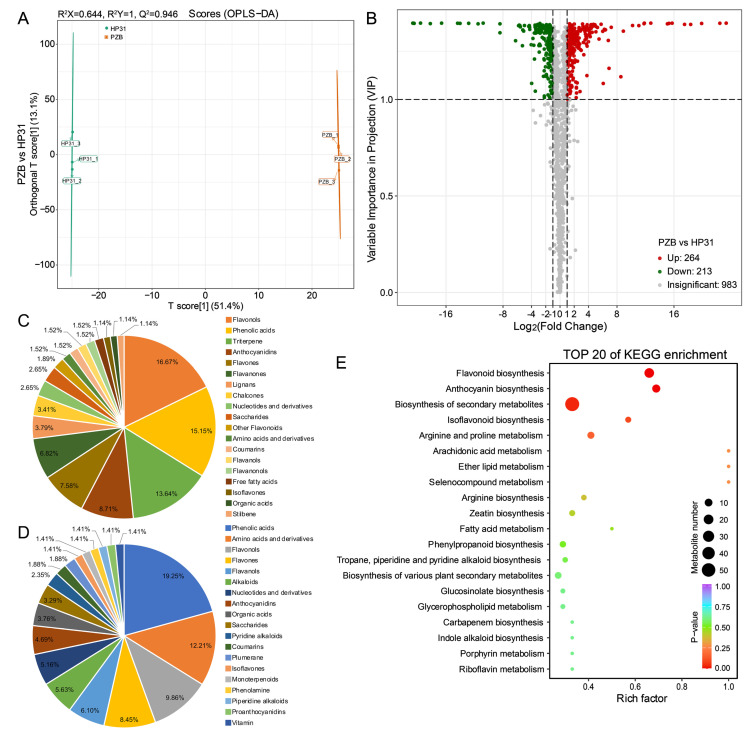
Overall analysis of the metabolomics data between dark-red and yellow fruits of Chinese cherry. (**A**) OPLS-DA. (**B**) Volcano plots of the metabolic profile in dark-red and yellow fruits. (**C**,**D**) Type and number of up-regulated and down-regulated metabolites. The TOP 19 types of DEMs were shown here. (**E**) TOP 20 of KEGG enrichment in the differential metabolites.

To further confirm the key differential expressed metabolites (DEMs) in anthocyanin biosynthesis pathway between dark-red and yellow fruits, we obtained the metabolic profiling of ‘HP31’ and ‘PZB’ fruits at mature stage (S5) using liquid chromatography tandem mass spectrometry (LC-MS/MS). The raw data has been deposited to the MetaboLights database under accession number MTBLS6752. The principal component analysis (PCA) results revealed significant differences between them (Supplementary Figure S8). The two principal components, PC1 and PC2, were 44.35% and 11.50% in the fruits, respectively. Based on orthogonal partial least squares discriminant analysis (OPLS-DA), the *Q^2^Y* value was 1, supporting the reliability of the metabolomic data (Figure 5A).

A total of 1460 metabolites in the fruits were identified by using UPLC-MS/MS (Supplementary Figure S8). The most abundant metabolites were flavonoids (380, 26.03%), followed by phenolic acids (243, 16.64%), others (142, 9.73%), amino acids and their derivatives (123, 8.42%), and terpenoids (113, 7.74%) (Supplementary Figure S9). Five anthocyanins and three flavonols existed only in dark-red fruits, and three anthocyanins and five flavonols were specifically detected in yellow fruits (Supplementary Table S4). Based on thresholds (|log_2_fold change| ≥ 1, variable importance in projection (VIP) ≥ 1), we obtained 477 DEMs between dark-red and yellow fruits (Figure 5B). Among them, 264 metabolites were up-regulated and 213 metabolites were down-regulated in dark-red fruits. Notably, anthocyanidins, flavones, and flavanones were up-regulated, whereas flavanols were down-regulated in dark-red fruits (Figure 5C,D). The KEGG enrichment analysis revealed that “flavonoid biosynthesis” (19 metabolites, ko00941), “anthocyanin biosynthesis” (11 metabolites, ko00942), and “biosynthesis of secondary metabolites” (54 metabolites, ko01110) were significant (Figure 5E).

### 2.7. Identification and Comparison of Anthocyanin and Procyanidin Compounds

**Figure 6:**
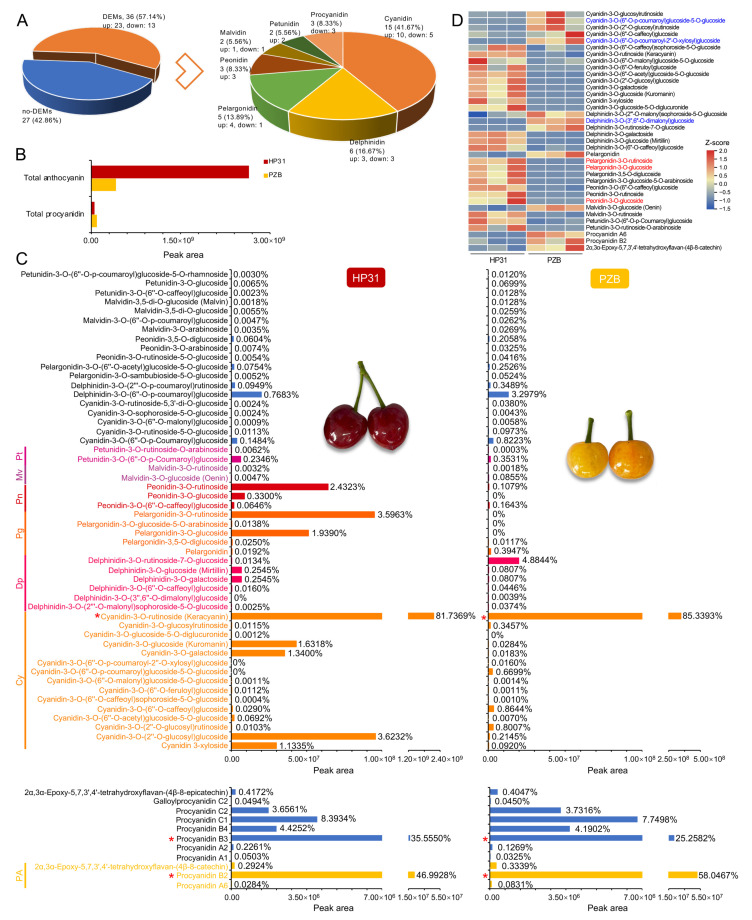
Comparison of anthocyanin and procyanidin compounds between dark-red and yellow fruits of Chinese cherry. (**A**) Type and number of differentially expressed metabolites. (**B**) The relative contents (peak area) of total anthocyanin and total procyanidin. (**C**) The relative contents and percentage of anthocyanin (upper) and procyanidin (lower) components in dark-red and yellow fruits of Chinese cherry. The blue font indicates the no-DEMs of procyanidins, and other colorful font indicates the 36 DEMs. Red asterisk indicates the predominant anthocyanin and procyanidin compounds. Abbreviations: Cy, cyanidin; Dp, delphinidin; Pg, pelargonidin; Pn, peonidin; Mv, malvidin; Pt, petunidin; PA, procyanidin. (**D**) Heatmap of the 36 DEMs. The color key from blue to red represents the relative content from −1.5 to 2.0. Red and blue font color indicates the top three up-regulated and down-regulated anthocyanins in the yellow vs. dark-red comparison.

A total of 52 anthocyanin compounds and 11 procyanidin compounds were identified from dark-red and yellow fruits (Supplementary Table S5). Among them, 36 DEMs were identified, including 33 anthocyanins (15 cyanidins, 6 delphinidins, 5 pelargonidins, 3 peonidins, 2 malvidins, and 2 petunidins), and 3 procyanidins (Figure 6A). The dark-red fruits accumulated more anthocyanin than yellow fruits, being 6.50-fold greater accumulation (Figure 6B). On the contrary, yellow fruits showed 1.84 times of total procyanidin in comparison to dark-red fruits (Figure 6B), among which procyanidin B2 (46.9928% in dark-red, 58.0467% in yellow) and B3 (35.5550% in dark-red, 25.2852% in yellow) were the major procyanidin compounds in both of them (Figure 6C).

In both dark-red and yellow Chinese cherry, cyanidin-3-*O*-rutinoside was the highest accumulated anthocyanin, accounting for 81.7369% and 85.3393% of the total anthocyanins, respectively, while it was 6.23-fold higher in dark-red than in yellow fruit (Figure 6C, Supplementary Table S5). Cyanidin-3-*O*-(2′′-*O*-glucosyl)glucoside, pelargonidin-3-*O*-rutinoside, and peonidin-3-*O*-rutinoside were also the major anthocyanin compounds (2.4323%~3.6232%) in dark-red fruits, while delphinidin-3-*O*-rutinoside-7-*O*-glucoside (4.8844%) and delphinidin-3-*O*-(6′′-*O*-*p*-coumaroyl)glucoside (3.2979%) were the second and third largest compounds in yellow fruit (Figure 6C). In the comparison, the greatest different metabolite was pelargonidin-3-*O*-rutinoside, being 1.05×10^7^-fold higher in dark-red than in yellow fruits (Figure 6C, Supplementary Table S5). The heatmap based on relative expression levels of the 36 DEMs showed that 23 anthocyanins were up-regulated, while 10 anthocyanins and 3 procyanidins were down-regulated in dark-red compared with yellow fruits (Figure 6D). Procyanidin B2 was 2.27 times in yellow fruits than that in dark-red fruits (Figure 6C). Therefore, it was obvious that the fruit color differences between dark-red and yellow fruits were not only determined by their total anthocyanin contents, but also by their anthocyanin components and percentages, especially cyanidin, pelargonidin, and peonidin derivatives, as well as the total procyanidin contents.

### 2.8. Comparison of Other Flavonoid Compounds in Flavonoid Pathway

To better understand the difference in flavonoid pathway between dark-red and yellow fruits, we further compared the type and number of other flavonoid compounds. The largest three sub-classes of flavonoids were flavonols (132, 34.74%), flavones (96, 25.26%), and flavanones (34, 8.95%) (Supplementary Table S5). In addition to anthocyanins, a total of 163 DEMs belonging to flavonoids were detected in the yellow vs. dark-red comparison, with 107 up-regulated and 56 down-regulated metabolites (Supplementary Figure S10). Among them, flavanones, flavanonols, and other flavonoids were all up-regulated, and the majority of DEMs from flavonols, flavones, and chalcones were up-regulated in dark-red fruits. Interestingly, 13 flavanol compounds were down-regulated among 17 differential expressed flavanols in dark-red fruits, suggesting that the accumulation of flavanol content was much higher in yellow fruits than in dark-red fruits (Supplementary Figure S10).

## 3. Discussion

### 3.1. Comparison of Anthocyanin and Procyanidin Compounds in Dark-red and Yellow Chinese Cherry Fruits

The fruit color is largely dependent on anthocyanins classes and their concentrations. Generally, pelargonidin is reportedly as indicating an orange-red color, while cyanidin and peonidin indicate a deep red or purplish-red color [42]. Cyanidin and its glycoside derivatives have been reported as the primary anthocyanins in red-colored cherries [21,26,43]. Cyanidin 3-*O*-rutinoside and cyanidin 3-*O*-glucoside are the major anthocyanin components in sweet cherry [24,44,45] and sour cherry fruits [46,47]. Pelargonidin 3-*O*-glucoside, cyanidin 3-*O*-rutinoside, and pelargonidin 3-*O*-rutinoside were the three dominant anthocyanin compounds in red tomentosa cherry [22,26]. Cyanidin-3-*O*-glucoside was the most abundant anthocyanin in Chinese dwarf cherry, followed by pelargonidin-3-*O*-glucoside [48,49]. In Chinese cherry, four cyanidin-based anthocyanins were detected, and cyanidin 3-rutinoside and cyanidin 3-glucosyl-rutinoside were the two major compounds for red fruits [22]. In the present study, a total of 52 anthocyanins belonging to 6 types were firstly isolated in both dark-red and yellow fruits, although their contents of major types were much lower in yellow fruits (Figure 6B,C). Cyanidin-3-*O*-rutinoside was the largest anthocyanin compound in both of them (>80%), generally similar to previous report [22], but it was 6.23-fold higher in dark-red than in yellow fruits. Meanwhile, two pelargonidins, one peonidin, and one cyanidin were also up-regulated in dark-red fruits, although their proportions were relatively low within total anthocyanin content (Figure 6C). Two delphinidin derivatives were also the important compounds in yellow fruits, accounting for 4.88% and 3.30% (Figure 6C). These results indicated that the color difference between dark-red and yellow Chinese cherry was not only dependent on the total anthocyanin content, but also on the anthocyanin components and proportion. In addition, our results further supported that the predominant anthocyanin compounds were similar among Chinese cherry, sweet cherry and sour cherry [22,23,24,25,44,46], while it was obvious different from tomentosa cherry [26] and Chinese dwarf cherry [48,49]. This was generally consistent with their traditional taxonomy classifications, being assigned into subgenus *Cerasus* and *Microcerasus* of genus *Cerasus*, respectively [15].

Procyanidin B type was predominant in cherry fruits as described by previous reports [25,26,50,51]. Procyanidin B2 and B4 accumulated from large green stage, decreasing their accumulation as the fruits ripened in sweet cherry cultivar ‘Lapins’ [52]. Procyanidin B2 was the major procyanidin (about 95%) in both red and white tomentosa cherry, while no significant difference was detected in total procyanidins between them [26]. The most abundant compounds in Chinese dwarf cherry genotypes were procyanidin B1 (24.54~48.79%) and B2 (4.90~20.35%) [50]. In this study, procyanidin B2 was the largest compound among 11 detected procyanidins in Chinese cherry, accounting for about half, followed by procyanidin B3 and C1 (Figure 6C). All the relative contents of procyanidin compounds were higher in yellow than dark-red fruits, with three up-regulated compounds (Figure 6B,C). Therefore, the greater procyanidins accumulation was also responsible for the light color of yellow fruits, strongly supported by the higher content of flavanol (the precursor of procyanidin) in yellow fruits (Supplementary Figure S10).

### 3.2. Key Candidate Genes Involved in Anthocyanin Biosynthesis of Chinese Cherry

**Figure 7:**
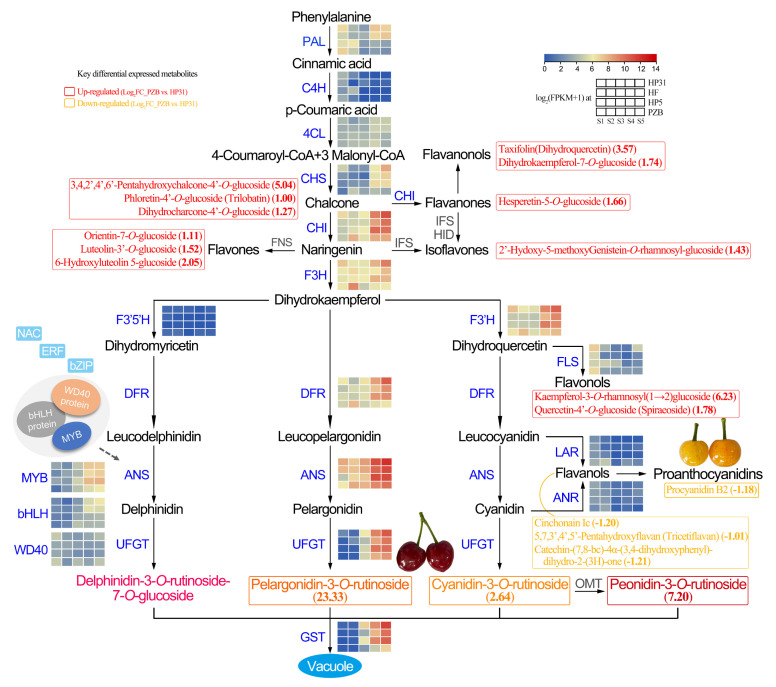
Regulatory network of anthocyanin biosynthesis in dark-red and yellow fruits of Chinese cherry. Grids represent the gene expression levels (log_2_(FPKM + 1) values): S1, S2, S3, S4, and S5, left to right. The red and yellow font represents the key up-regulated and down-regulated metabolites (log_2_fold change (PZB vs. HP31)). The black dotted line represents that the direct correlation was uncertain.

It has been widely reported that a series of structural genes, including *PAL*, *C4H*, *4CL*, *CHS*, *CHI*, *F3H*, *F3’H*, *DFR*, *ANS*/*LDOX*, and *UFGT*, co-regulated anthocyanin biosynthesis in many fruit crops. In the present study, a regulatory network of gene expression regulating anthocyanin biosynthesis and transport and the key differential expressed metabolites in Chinese cherry fruit was summarized, as shown in Figure 7. Combining the transcriptome data and RT-qPCR results, the up-regulation of structural genes (*CpCHS*, *CpCHI*, *CpF3H*, *CpF3’H*, *CpDFR*, *CpANS*, and *CpUFGT*) in (dark-red fruits enhanced flux in anthocyanin pathways. However, the low expression levels of these genes in yellow fruits generated a lack of stable anthocyanin synthesis. The DFR enzyme can selectively catalyze three kinds of substrates to synthesize three specific products: leucodelphinidin, leucopelargonidin, and leucocyanidin. The expression of F3’H promoted the synthesis of dihydroquercetin through DFR and UFGT, further forming cyanidin-3-*O*-rutinoside. Both the cyanidin and pelargonidin contents of dark-red fruits are considerably higher than that of yellow fruits (Figure 6C). Thus, the high expression of *CpF3’H* and *CpDFR* determined the synthesis of specific anthocyanin component, which is consistent with the anthocyanin biosynthesis in grape [53]. Among these genes, *CpANS* showed the highest expression level, and *CpF3H* and *CpUFGT* exhibited the biggest difference (Figure 4). This suggested that the above seven genes are potential key genes regulating anthocyanin biosynthesis in Chinese cherry, especially *CpF3H*, *CpANS*, and *CpUFGT*. This was largely consistent with the results in many other Rosaceae fruit crops such as sweet cherry [27,28], tomentosa cherry [26], and peach [54].

GSTs are known to participate in the anthocyanin transport and accumulation, while the absence of GSTs often results in an anthocyanin-less phenotype with reduced pigmentation [7,8]. Here, four GST genes significantly increased in dark-red fruits compared to those in yellow fruits (Figure 3). Therefore, the upregulation of *CpGST* played a vital role in anthocyanin transport and resulted in the accumulation in vacuoles. Interestingly, the expression level of *CpGST* was about 8.91-fold greater at S5 than S4 stage in yellow fruits (Figure 4), which generated yellow with blush at mature stage (Figure 1A). This suggested that the anthocyanin biosynthesis pathway was fluent in yellow fruits, but the reduction in precursors finally resulted in the less accumulation of anthocyanins due to the lower expression levels of EBGs (*CpCHS*, *CpCHI*, *CpF3H*, and *CpF3’H*).

LAR and ANR are regarded as two key enzymes in procyanidin biosynthesis. As reported in *Medicago truncatula*, The loss of function of LAR in seed coats decreased the levels of procyanidins [55]. The higher expression level of *CpLAR* in yellow fruits (Figure 4) probably contribute to the more accumulated flavanols from the early stage, finally forming more procyanidins at mature stage (Figure 6B,C). It has been reported that competition existed between anthocyanins and flavonols biosynthesis in red apple fruits during fruit ripening [56], and apple flowers [57]. Higher amount flavonols were detected for all stages of white flower development than red flower in peach [58]. However, this study exhibited a different branch to flavanols and procyanidins rather than flavonols in bicolored (yellow) fruits in Rosaceae family.

### 3.3. Transcription Factors Involved in Anthocyanin Biosynthesis of Chinese Cherry

Anthocyanin metabolism is also regulated by a series of transcription factors, such as MBW (MYB-bHLH-WD40) protein complex, NAC, WRKY, ERF, and bZIP families. MYB TFs are reportedly associated with the regulation of anthocyanin biosynthesis and accumulation in sweet cherry [29], apple [7], and blackberry [59]. bHLH commonly interacts with MYB and WD40 to regulate anthocyanin biosynthesis jointly [60]. Based on to the expression levels, we identified seven MYB and six bHLH TFs (Figure 3B). Anthocyanin biosynthesis has been proven to be positively regulated by MYB TFs, such as *MYB10* [29,61,62], *MYB20* [63], and *MYB306* [64], by bounding to the promoter of structural genes or interacting with bHLH genes. *MYB4*, a repressor activated by *bHLH3*, prevents the formation of the MBW through competitive binding with *bHLH3* to inhibit the accumulation of anthocyanins by down-regulation of CHS, ANS, and DFR in mulberry [65], and bananas [66]. In addition, *bHLH148* and *bHLH10* were specifically expressed in yellow Chinese cherry (Figure 3B), suggesting their possible negative role in regulating anthocyanin biosynthesis. A WD40 protein, homologous to Arabidopsis TTG1 [67], was identified, which has also been characterized from apple [68] and strawberry [69]. The expression level of WD40 was significantly higher in yellow than dark-red fruits (Figure 3B), indicating its negative regulating role. Therefore, the anthocyanin biosynthesis in Chinese cherry fruit is regulated by the relevant MBW protein complex (Figure 7).

We also obtained two NAC, two WRKY, one ERF, and two bZIP that were significantly upregulated in dark-red fruits. These TFs have been proven to directly or indirectly regulate anthocyanin biosynthesis by binding to an MYB promoter or through protein-protein interactions in other fruits [35,37,38,39]. For example, *MdNAC52* can bind to the promoters of *MdMYB9* and *MdMYB11* to increase the anthocyanin content by regulating *MdLAR* in apple [70]. The interaction of *PyWRKY26* and *PybHLH3* could co-target the *PyMYB114* promoter, which resulted in anthocyanin accumulation in red-skinned pear [41]. *MdbZIP44* enhances *MdMYB1* binding to downstream target gene promoters to promote anthocyanin biosynthesis in apple [39]. Overall, eight regulatory genes were confirmed as determinants of fruit color in Chinese cherry. Our findings can enrich the key candidate genes and metabolites involved in the anthocyanins biosynthesis in Chinese cherry fruits, which are of great importance for molecular marker-assisted breeding.

## 4. Materials and Methods

### 4.1. Plant Materials

A total of four Chinese cherry accessions including ‘HP31’ (dark-red), ‘HF’ (red), ‘HP5’ (light-red) and ‘PZB’ (yellow) were grown under field conditions at the cherry germplasm resources of Sichuan Province (Chengdu City), China. Full flowering was set at 0 days after full bloom (DAFB) when 50% of flowers were open in the trees. Fruit samples were collected based on fruit phenology (Table 1). For ‘HP31’, the sampling was: green (S1), 37 DAFB; light green (S2), 41 DAFB; pink blush (S3), 46 DAFB; red (S4), 51 DAFB; dark red (S5), 55 DAFB. For ‘HF’, the sampling was: green (S1), 37 DAFB; light green (S2), 43 DAFB; pink blush (S3), 46 DAFB; light red (S4), 49 D AFB; red (S5), 53 DAFB. For ‘HP5’, the sampling was: green (S1), 36 DAFB; light green (S2), 40 DAFB; pink blush (S3), 45 DAFB; orange red (S4), 50 DAFB; light red (S5), 54 DAFB. For ‘PZB’, the sampling was: green (S1), 37 DAFB; light green (S2), 43 DAFB; straw yellow (S3), 48 DAFB; yellow (S4), 52 DAFB; yellow with blush (S5), 58 DAFB (Supplementary Table S6). Fruits were collected for their uniform size, same appearance, and no defects. Ten cherries were analyzed to measure their color parameter, and the other thirty fruits were immediately frozen in liquid nitrogen and stored at −80℃ for subsequent analysis.

### 4.2. Fruit Color Assessment

The fruit peel color was measured using a HunterLab chromameter (Konica Minolta, Inc., Tokyo, Japan) according to the CIE system. Positive a* values indicated red and purple, and negative values indicated green and blue. Positive b* values represented yellow, and negative values represented blue. Three sets of a* and b* values were measured for the equatorial part of each fruit and used to calculate the color ratio (a*/b*) [32]. Three biological replicates per sample point were analyzed, with ten cherries for each replicate.

### 4.3. Total Anthocyanin and Flavonoid Content Measurement

The extraction and measurement of total anthocyanin content was conducted using a pH differential method [71]. About 1.5 g fruit was extracted with 15 mL of extraction solution (acetone:methanol:water:acetic acid = 2:2:1:0.5), after a water bath at 40 °C, the mixture was centrifuged at 8,000 g for 25 min, and the supernatant was used for determination. Two buffer systems were employed, with 0.4 M potassium chloride (pH 1.0) and 0.4 M dibasic sodium (pH 4.5). Total anthocyanin content was calculated according to the equation: A = [(A_510_ − A_700_) _pH 1.0_ − (A_510_ − A_700_) _pH 4.5_], which was converted into mg cyanidin 3-glucoside per 1,000 g fresh weight (FW). Three independent biological replicates per sample point were analyzed.

Aluminum chloride method was utilized for measurement of the total flavonoid content [72]. Stock solution was prepared by dissolving 50 mg quercetin in 50 mL of methanol. About 5 mg fruit extracts were mixed in 5 mL of distilled water and 0.3 mL of 5% NaNO_2_. 0.6 mL of 10% AlCl_3_ and 2 mL of 1.0 M NaOH were combined in the above solution after 5 min. The absorbance of the reaction mixture was measured at 510 nm using a spectrophotometer. Total flavonoid content was calculated as quercetin equivalents (mg/g FW), and performed in triplicates.

### 4.4. Transcriptome Analysis

Three biological replicates with mixed three fruits each, including fruit peel and flesh, were used for RNA-seq. A total of 1 μg RNA per sample was used for RNA preparations. The mRNA molecules were purified using oligo(dT)-attached magnetic beads and then were fragmented into small pieces using fragmentation reagent. The first-strand and second-strand cDNAs were synthesized using random hexamer-primed reverse transcription. End repair, polyadenylation, adapter ligation, PCR amplification, and library quality control were carried out following the DNABSEQ RNA-Seq library preparation protocol. A total of 60 libraries were sequences on an MGI2000 platform to generate raw 150 bp paired-end reads. For downstream analyses, high-quality clean reads were obtained by filtering low-quality reads and those containing adapters or poly-N in SOAPnuke software (−n 0.01–1.20, −q 0.4–A 0.25-cutAdaptor).

The reference genome database and gene annotation files were extracted from Chinese cherry (unpublished data). Clean reads were mapped onto the reference genome using Hisat2 v.2.1.0. The read counts and FPKM values for each gene were calculated in StringTie v.1.3.5. The DEGs were identified using EdegR package with the screening conditions |log_2_fold change| ≥ 2 and DFR ≤ 0.01. Structural and regulatory genes involved in anthocyanin biosynthesis pathway were screened from DEGs.

WGCNA was conducted using the WGCNA R package with default settings (v1.4.1717). All genes were imported into WGCNA to construct co-expression modules using the automatic network construction function block-wise Modules. Correlations between modules and color ratio, and anthocyanin and flavonoid content at each developmental stage were analyzed with respect to all genes in each module. Significant trait-related modules were identified based on high correlation values. Using default settings, genes from the MEblack module were exported for Cytoscape software (v.3.9.1) [73].

### 4.5. Real-Time PCR Analysis

The expression levels of nine structural genes and eight transcriptional factors in the anthocyanin biosynthesis pathway were determined by RT-qPCR. The gene-specific primers were designed by Primer 5.0 and shown in Supplementary Table S7. Total RNA was extracted from the fruits at different developmental stages using the Plant Total RNA Isolation Kit (SK8631; Sangon Biotech, Shanghai, China). The cDNA was synthesized from RNA using the PrimeScriptTM RT-PCR Kit (RR047A; TaKaRa Bio, Kusatsu, Japan). The RT-qPCR was performed in a 20 μL reaction volume using the TransStart^®^ Green qPCR SuperMix (TransGen Biotech Co., Ltd., Beijing, China) on a CFX96 TouchTM Real-Time PCR detection system (Bio-Rad, Hercules, CA, USA). The reaction procedure is as follows: 95 °C for 30 s, followed by 40 cycles of 95 °C for 5 s, 60 °C for 30 s, and 72 °C for 30 s. The 2^−ΔΔCT^ method was used to calculate the gene expression levels with the geometric mean of the two housekeeping genes (cherry actin and ubiquitin). Three independent biological replicates were analyzed for each sample point.

### 4.6. Metabolome Analysis

The freeze-dried matured cherry samples with mixed peel and flesh were crushed using a mill (MM400, Retsch, Germany) with a zirconia bread at 30 Hz for 1.5 min. Approximately 50 mg of samples was extracted overnight at 4 °C with 1.2 mL of 70% methanol before performing centrifugation at 12,000 rpm for 3 min. The supernatants were pooled and filtered with a microporous membrane (0.22 μm). The relative quantification of widely targeted metabolites in Chinese cherry fruit using an UPLC-ESI-MS/MS system (UPLC, ExionLC^TM^ AD; MS, Applied Biosystems 6500 Q TRAP, https://sciex.com.cn/, accessed on 21 October 2022). Quantification of metabolites was carried out using a scheduled multiple reaction monitoring method. Metabolite profiling and metabolomics data analyses were conducted by Metware Biotechnology Co., Ltd. (Wuhan, China).

PCA and OPLS-DA were conducted to verify the differences and reliability of metabolites. DEMs were determined by a VIP ≥ 1 and absolute log_2_fold change (≥1). Then the DEMs were mapped to the KEGG database and their significance was determined by hypergeometric test’s *p*-values.

### 4.7. Statistical Analysis

The data was analyzed using IBM SPSS Statistics software (v25.0). The results were expressed as mean ± standard deviation (SD). A *p* ≤ 0.05 was considered a statistically significant difference (Tukey’s test).

## 5. Conclusions

In summary, this is the first study to investigate the coloring patterns and the corresponding accumulation of anthocyanin between dark-red and yellow Chinese cherry fruits. Based on LC-MS/MS, we identified 33 and 3 differential expressed metabolites related to anthocyanins and proanthocyanidins between mature dark-red and yellow fruits. The anthocyanins were mainly up-regulated, while the proanthocyanidins were all down-regulated in dark-red fruits. By transcriptome analysis, eight biosynthesis genes (*CpCHS*, *CpCHI*, *CpF3H*, *CpF3’H*, *CpDFR*, *CpANS*, *CpUFGT*, and *CpGST*) were significantly more highly expressed in dark-red fruits, especially *CpANS*, *CpUFGT*, and *CpGST*. *CpLAR* was higher in yellow fruits than dark-red fruits, especially at the early stage. Eight regulatory genes (*CpMYB4*, *CpMYB10*, *CpMYB20*, *CpMYB306*, *bHLH1*, *CpNAC10*, *CpERF106*, and *CpbZIP4*) were also identified as determinants of fruit color in Chinese cherry. These findings can enrich the key genes and metabolites involved in the anthocyanins biosynthesis in Chinese cherry, which are of great importance for molecular marker-assisted breeding.

## Figures and Tables

**Table 1:** Division of fruit phenology of Chinese cherry with different fruit color.

Fruit Phenology	Stage	Color Difference	Fruit Color		
			Dark-Red	Red/Light-Red	Yellow
Green ripening period	S1	a*	−11.34~−7.74	−10.56~−6.77	−10.00
		b*	43.13~44.35	40.11~45.75	37.63
		a*/b*	−0.26~−0.17	−0.26~−0.14	−0.27
Color conversion period	S2	a*	−7.98~−5.85	−8.21~1.08	−9.62
		b*	46.24~46.85	42.32~47.66	38.32
		a*/b*	−0.17~−0.12	−0.19~0.15	−0.25
	S3	a*	5.27~16.74	2.25~10.29	−1.66
		b*	33.80~40.28	31.02~43.00	35.72
		a*/b*	0.13~0.52	0.05~0.26	−0.05
Fruit ripening period	S4	a*	26.54~28.82	24.43~27.09	6.31
		b*	21.91~23.48	12.27~26.70	24.07
		a*/b*	1.22~1.23	0.93~2.20	0.26
	S5	a*	20.43~21.13	22.16~23.22	8.95
		b*	7.27~8.24	8.82~16.58	20.57
		a*/b*	2.55~2.98	1.68~2.53	0.43

Note: The standard was summarized based on eight representative Chinese cherry accessions with different fruit color.

## Data Availability

Not applicable.

## Supplementary Materials

The supporting information can be downloaded at: https://www.mdpi.com/article/10.3390/ijms24043471/s1.
