# Supplementary material for: Integrated Transcriptome and Metabolome Analyses Provide Insights into the Coloring Mechanism of Dark-red and Yellow Fruits in Chinese Cherry [Cerasus pseudocerasus (Lindl.) G. Don]
Source: Int J Mol Sci. 2023 Feb 9;24(4):3471. doi: 10.3390/ijms24043471 (PMC9965709; doi:10.3390/ijms24043471)
Supplement: Supplementary file 1 [file ijms-24-03471-s001.zip › Proof Supplementary Figures 20230208.pdf]

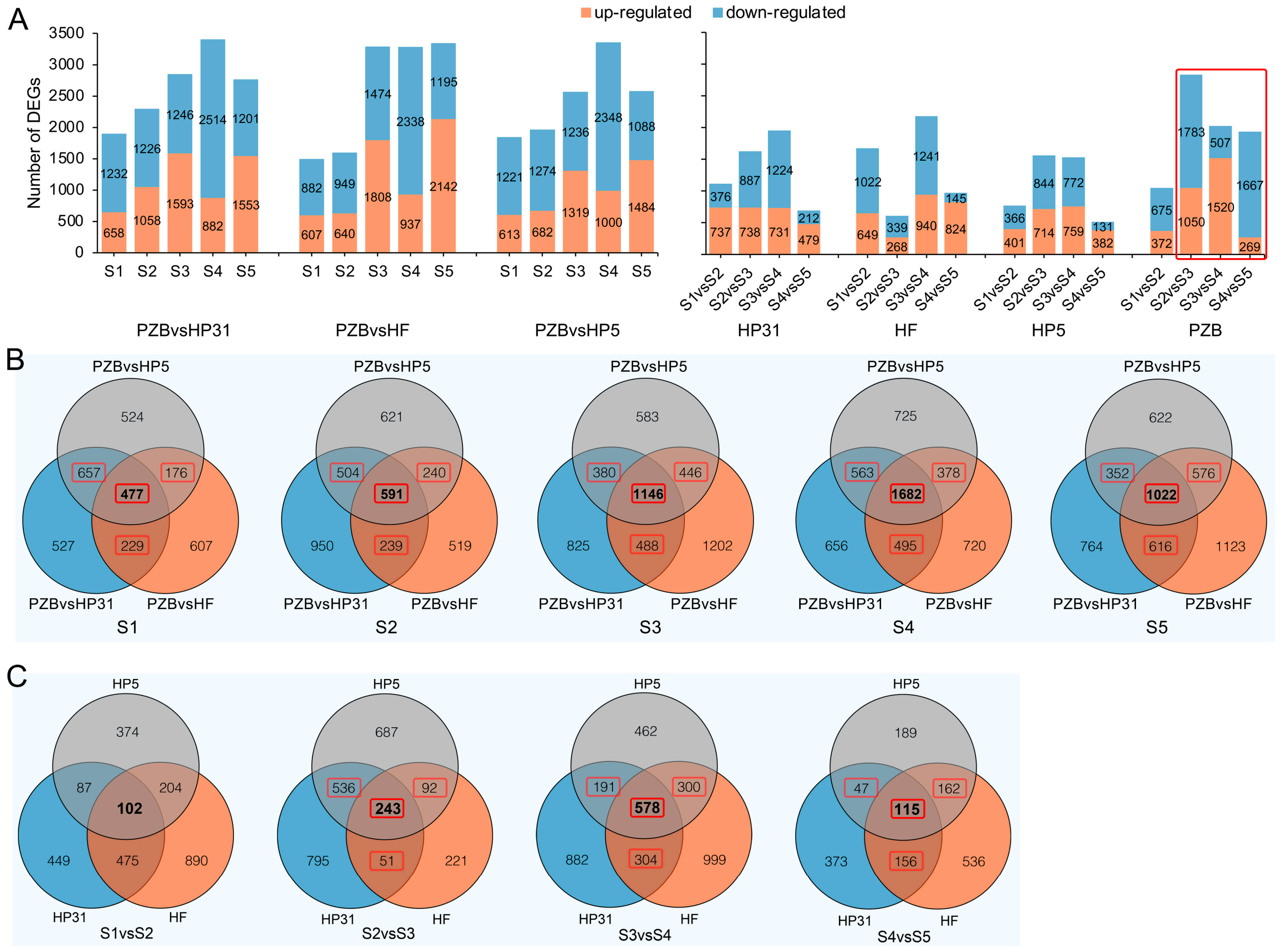

Supplementary Figure S1. The differentially expressed genes analysis. (**A**) The number of DEGs in pairwise comparison groups. (**B**) Venn diagrams of DEGs between dark-red and yellow fruits at S1−S5 stage respectively. (**C**) Venn diagrams of DEGs during developmental stage within dark-red fruits.

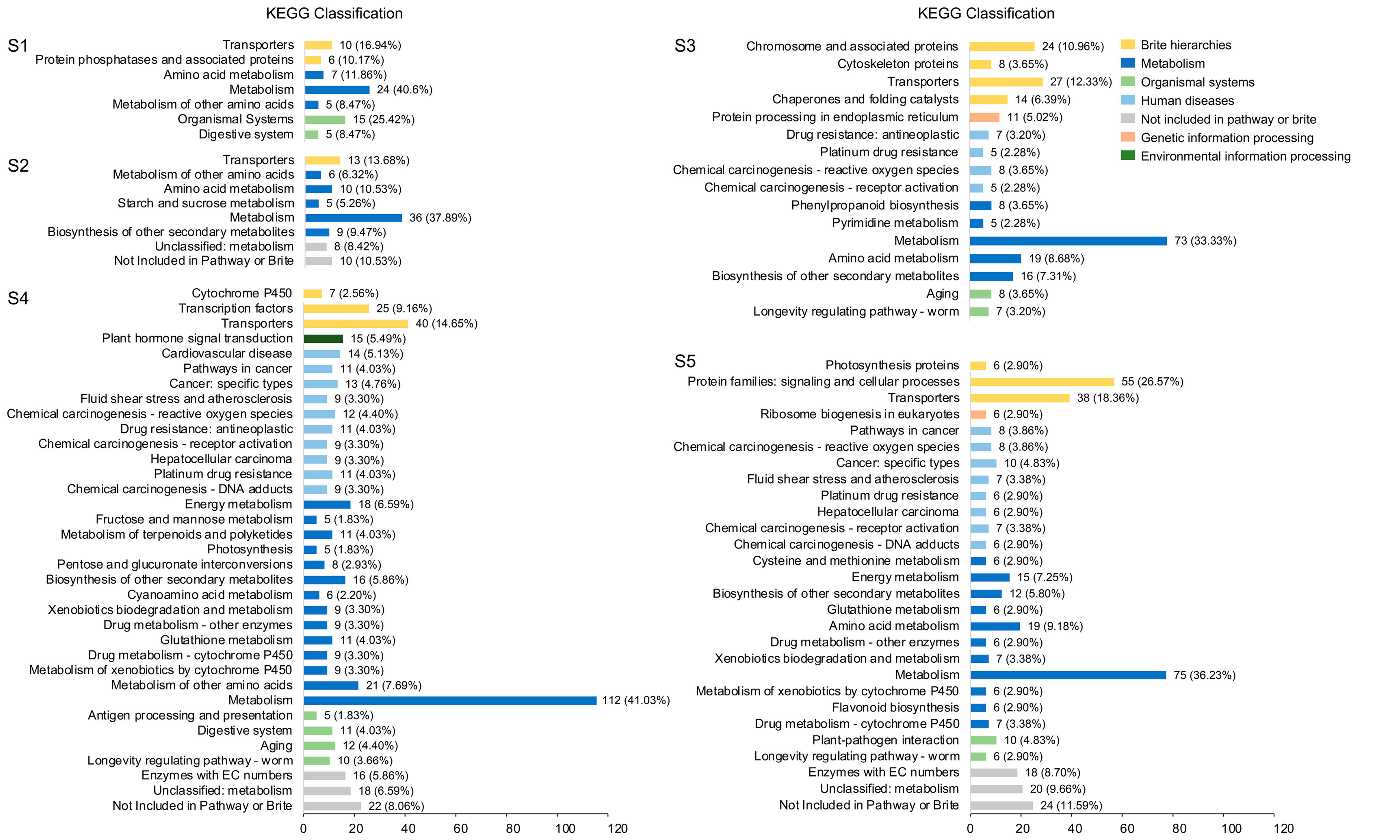

Supplementary Figure S2. KEGG classification in the common DEGs between dark-red and yellow fruits at five developmental stages. A total of 477, 591, 1146, 1682, and 1022 common DEGs from the venn diagrams between dark-red and yellow fruits at S1−S5 stages were used for analysis.

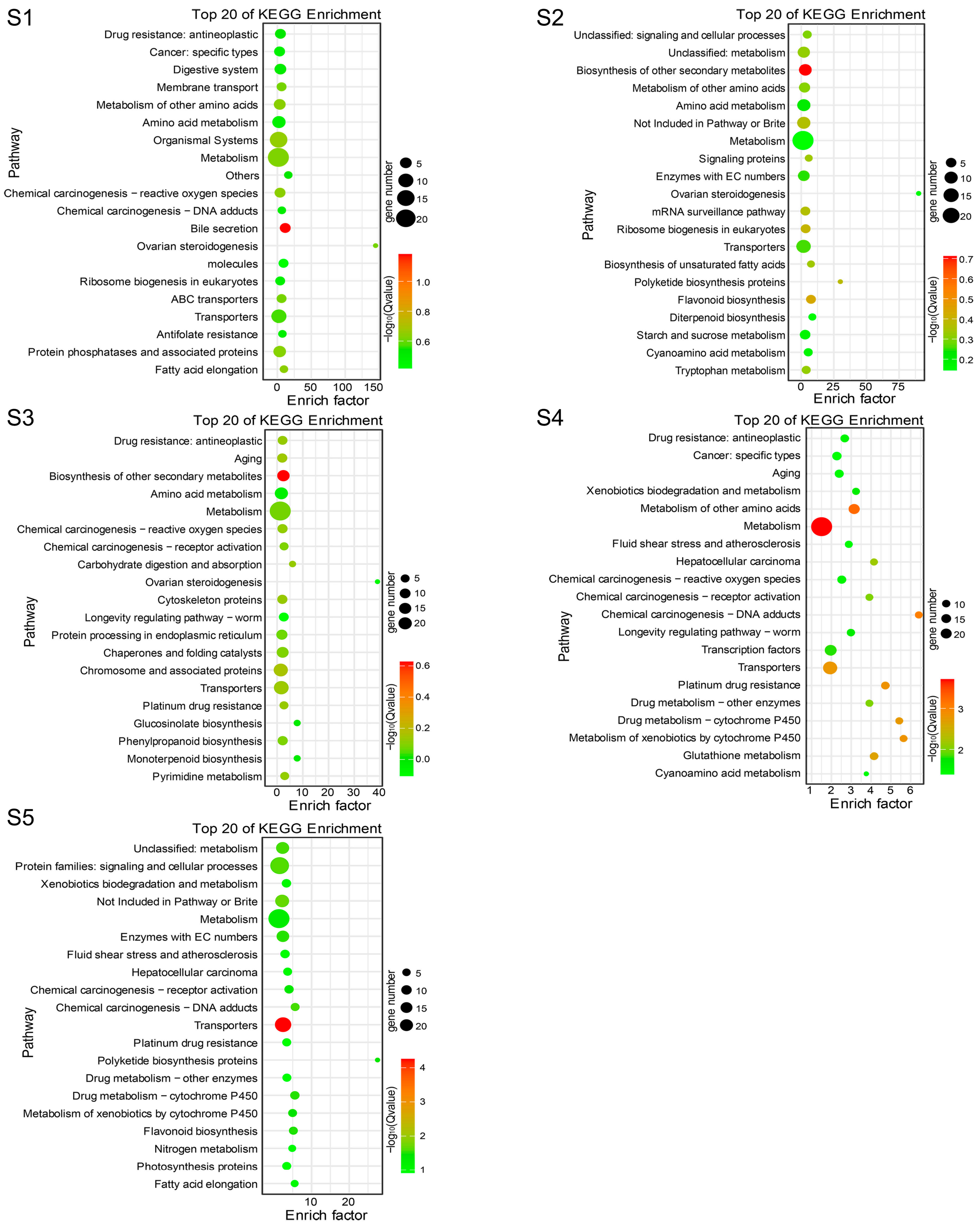

Supplementary Figure S3. KEGG enrichment analysis with the top 20 KEGG pathways in the common DEGs between dark-red and yellow fruits at five developmental stages. A total of 477, 591, 1146, 1682, and 1022 common DEGs from the venn diagrams between dark-red and yellow fruits at S1-S5 stages were used for KEGG enrichment analysis.

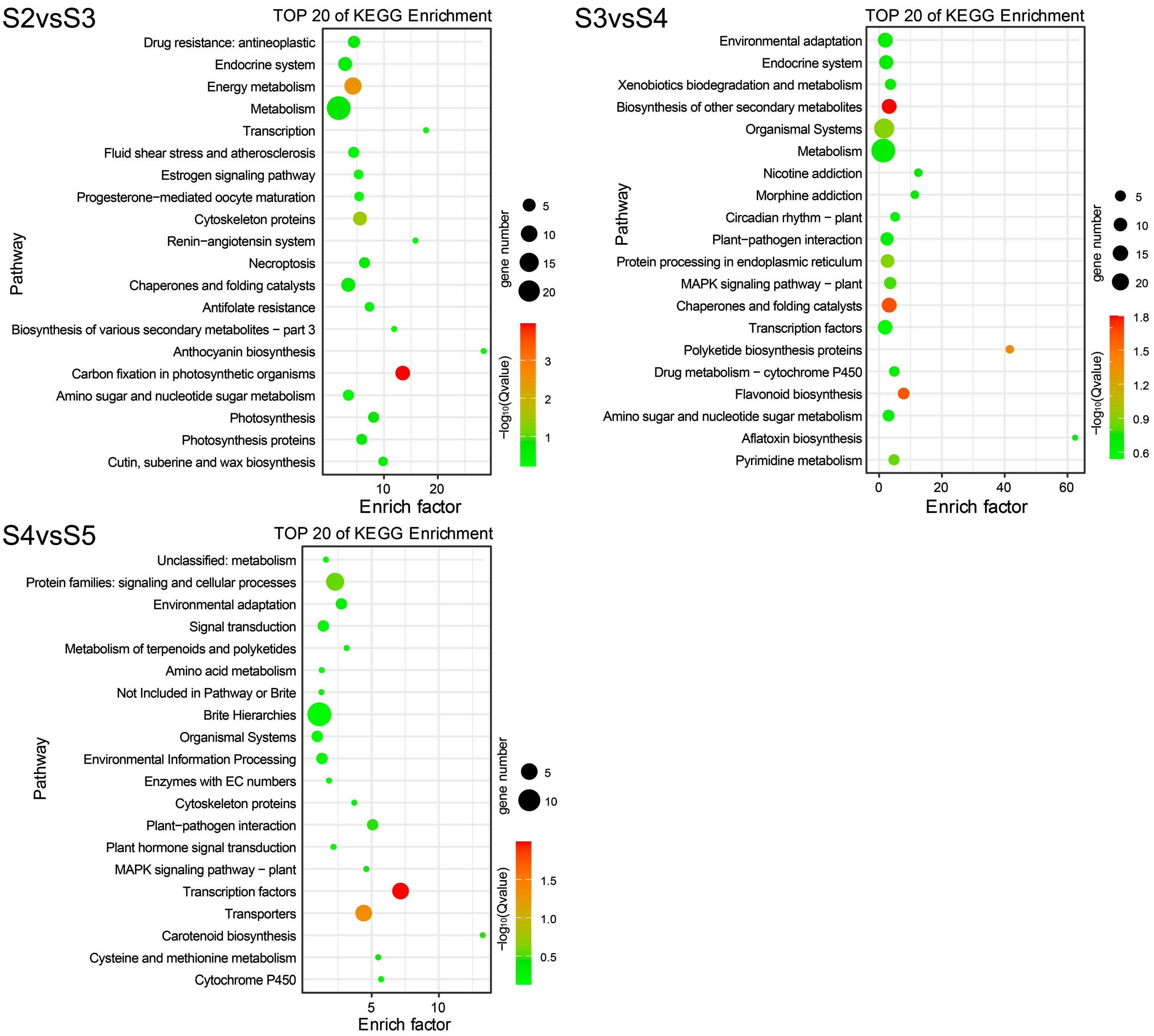

Supplementary Figure S4. KEGG enrichment analysis with the top 20 KEGG pathways in the common DEGs during fruit development within dark-red fruits. A total of 243, 578, and 115 common DEGs from the venn diagrams during developmental stage within red fruits were used for KEGG enrichment analysis.

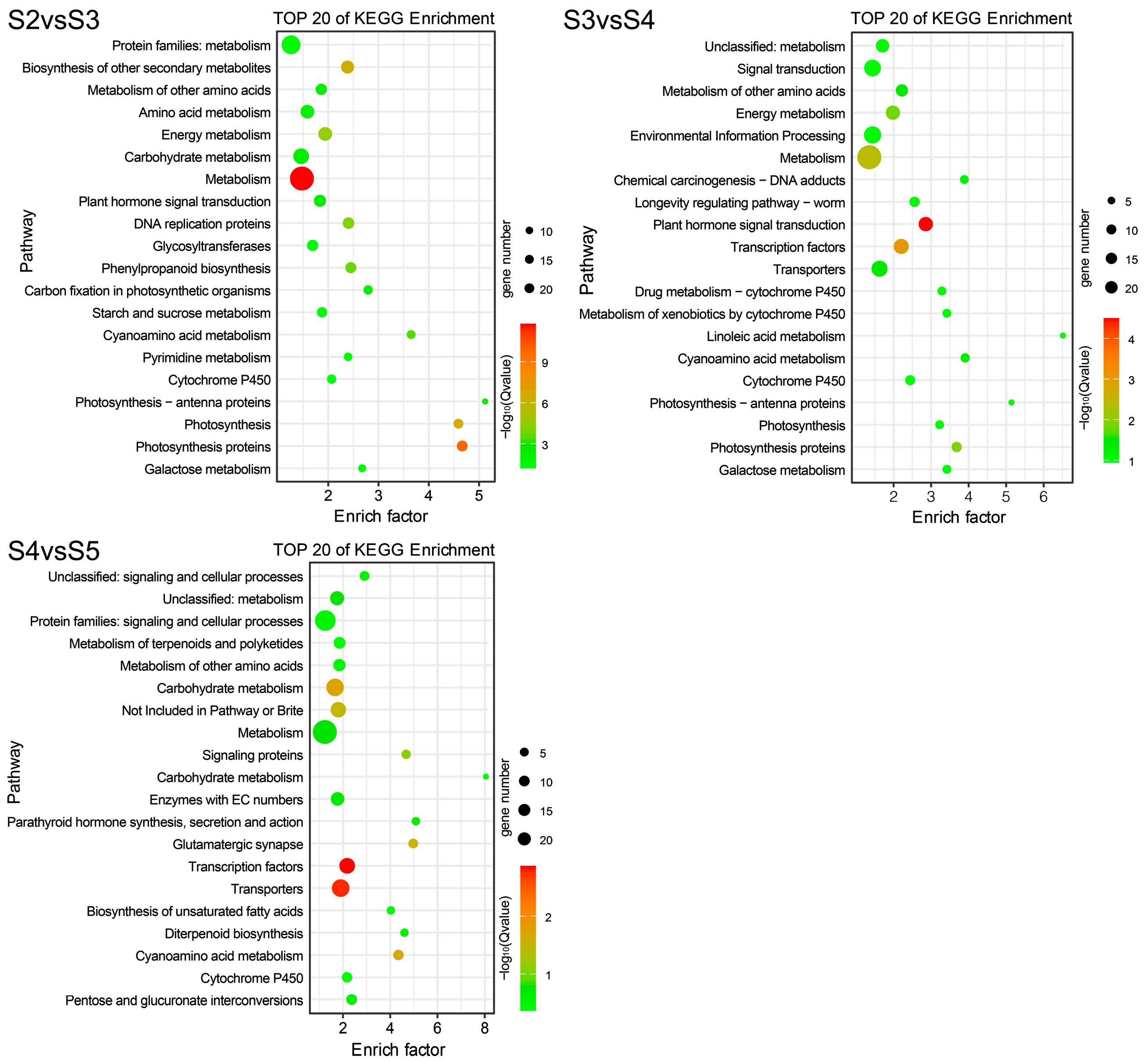

Supplementary Figure S5. KEGG enrichment analysis with the top 20 KEGG pathways in the DEGs during fruit development in yellow fruits. A total of 2833, 2027, and 1936 DEGs during developmental stage in yellow fruits were used for KEGG enrichment analysis.

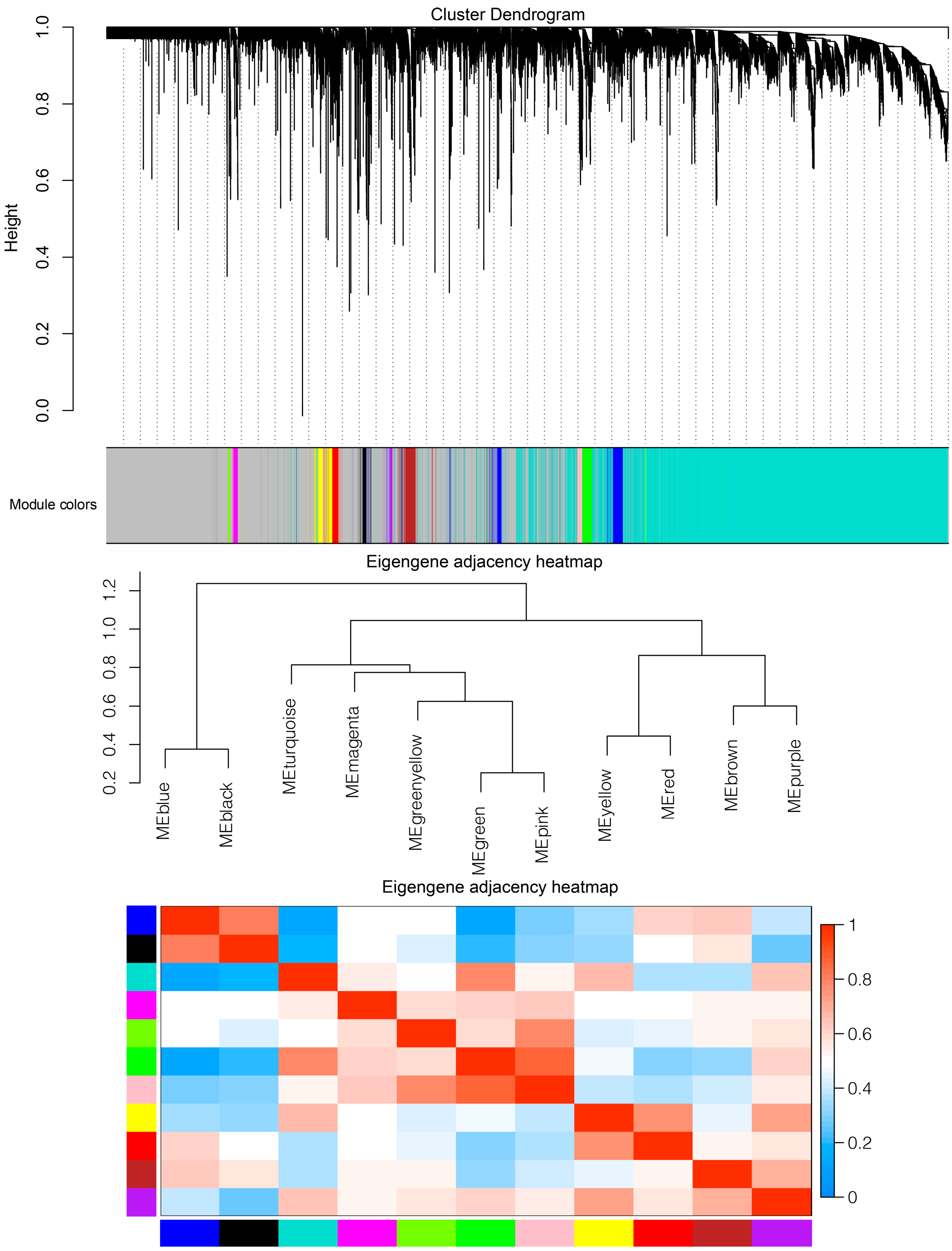

Supplementary Figure S6. Hierarchical clustering dendrogram of module and module eigengenes and heatmap plot of the adjacencies in the eigengene network.

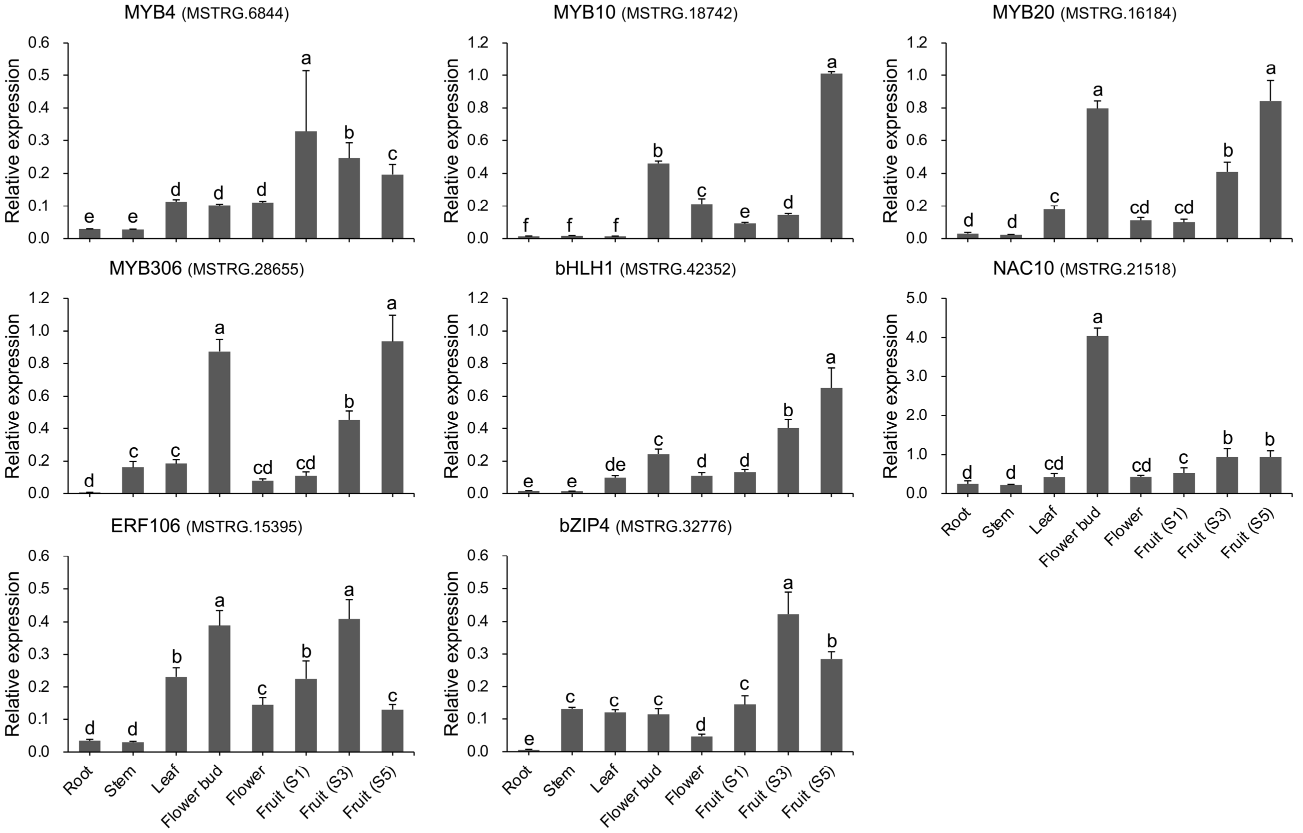

Supplementary Figure S7. Expression analysis of transcription factors related to the anthocyanin biosynthesis pathway in different tissues of Chinese cherry. The root, stem, leaf, flower bud (red), flower (pinkish), fruit (S1, S3, and S5) were all from HF (Hongfei). Error bars indicated ± SD from three independent replicates. The lower case letter indicate significant difference at 0.05 level.

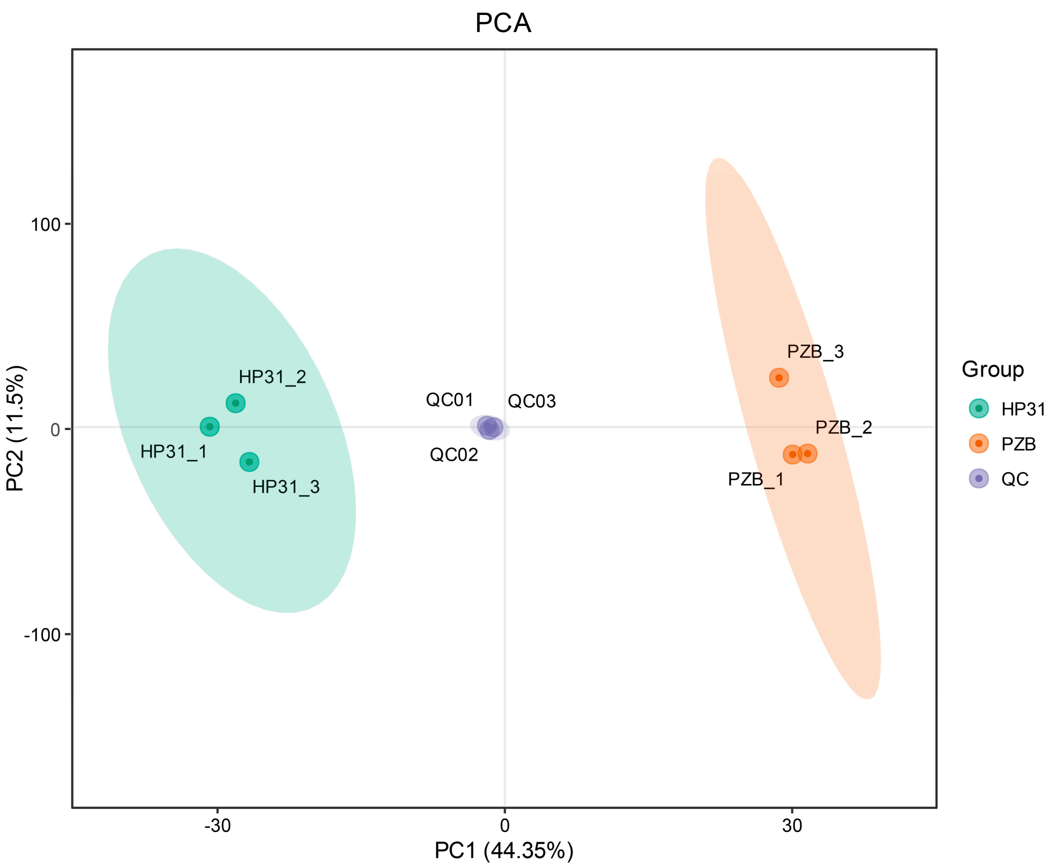

Supplementary Figure S8. Principal components analysis (PCA) of all samples based on metabolic profiling.

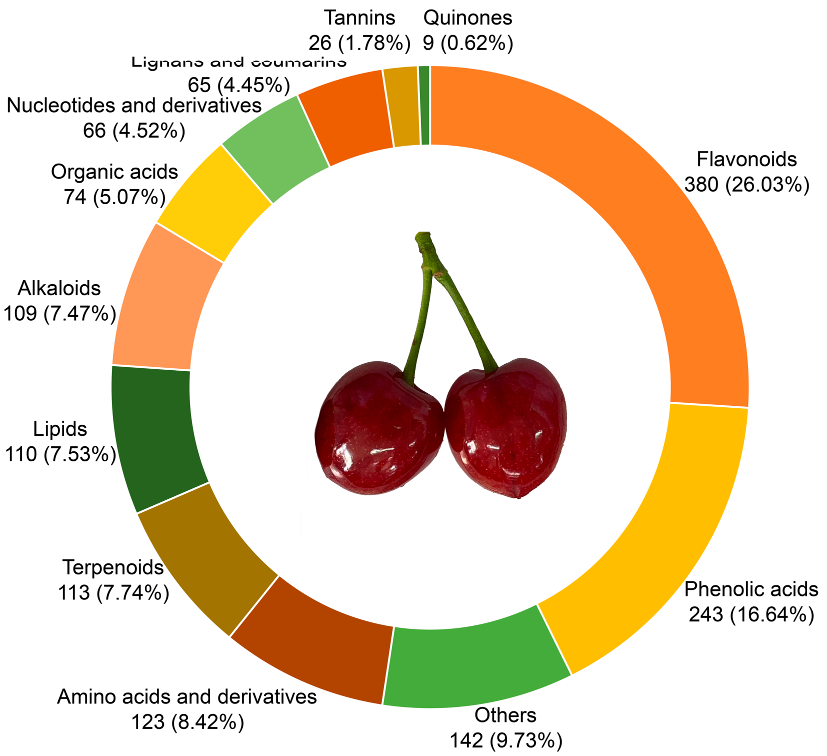

Supplementary Figure S9. Type and number of metabolites in Chinese cherry fruits.

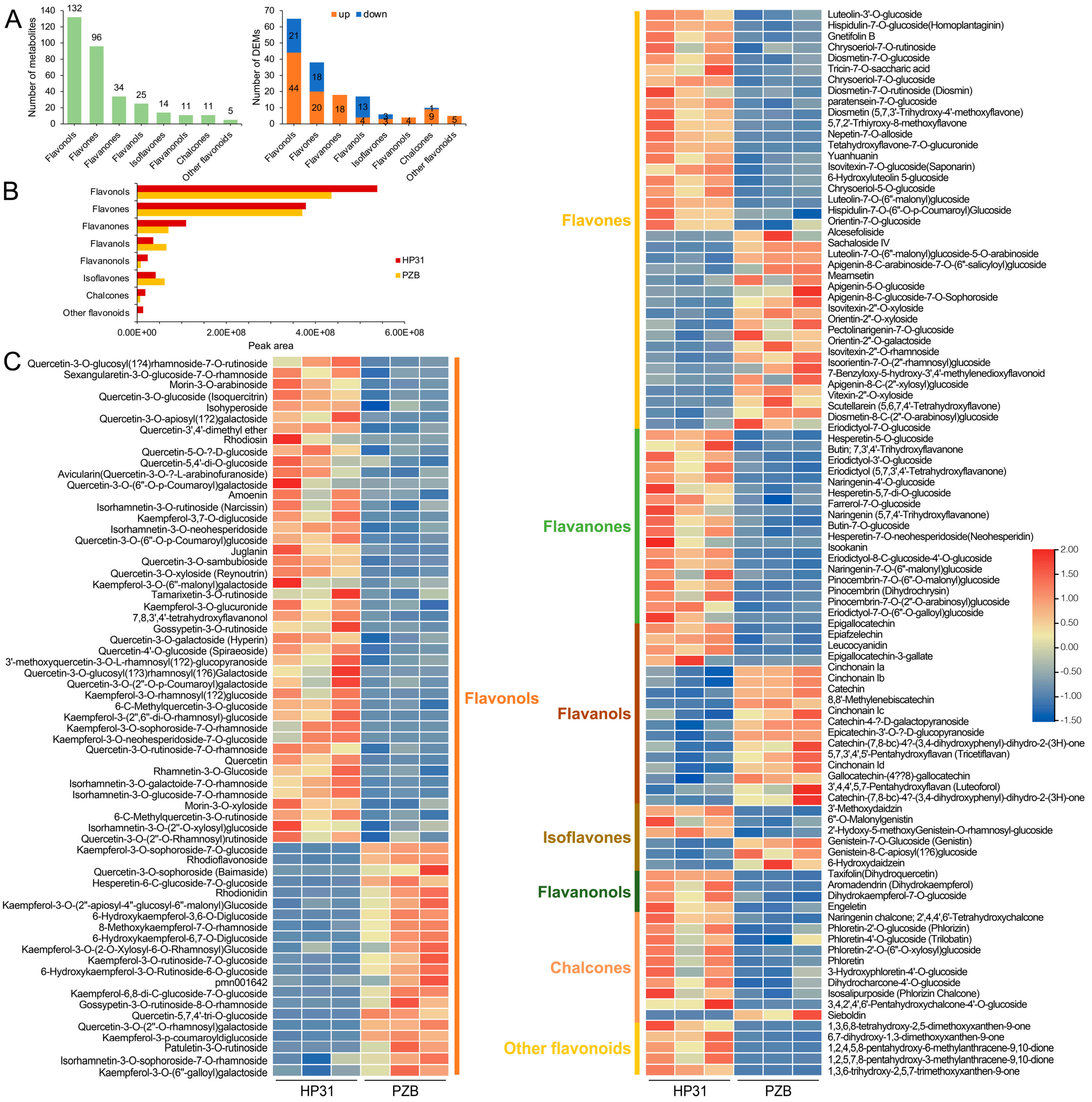

Supplementary Figure S10. Comparison of flavonoid compounds between dark-red and yellow fruits of Chinese cherry. (**A**) Statistics of the expressed metabolites and differentially expressed metabolites. (**B**) The relative contents of flavonoid compounds from metabolome data. (**C**) Heat map of the 163 DEMs (anthocyanins excluded) at mature stage. Color indicates the relative content of each DEM, from blue (low) to red (high).
